# The preparatory process of the 2023 Mw 7.8 Türkiye earthquake

**DOI:** 10.1038/s41598-023-45073-8

**Published:** 2023-10-19

**Authors:** Matteo Picozzi, Antonio G. Iaccarino, Daniele Spallarossa

**Affiliations:** 1 University of Naples Federico II, Naples, Italy; 2 DISTAV, University of Genoa, Genoa, Italy

**Keywords:** Seismology, Natural hazards

## Abstract

To verify the existence of a preparatory process for the 6 February 2023, Mw 7.8 Kahramanmaraş earthquake, southern Türkiye, we analyze the temporal evolution of seismic catalog information for ~ 7500 earthquakes with magnitudes M_L_ ≥ 1.5, which occurred along the main segments of the East Anatolian Fault (EAF) since 2014. We find the EAF fault segments showing different temporal patterns in the proportion of nonclustered seismicity, which we interpret as temporal variation of coupling. We also study the evolution of the *b*-value, fractal dimension and energy rate. These seismic features show for the Amanos and Pazarcık fault segments a long-term trend during the period 2020–2022 that might correspond to a quiescence phase. The latter is followed by a change in earthquakes clustering and characteristics that starts about eight months before the Mw 7.8 Kahramanmaraş event. Our observations confirm the existence of a long-lasting preparatory phase for the 2023, Mw 7.8 Kahramanmaraş earthquake and can stimulate new investigations on the East Anatolian Fault mechanic. Intercepting when a fault starts deviating from its steady behavior, might be the key for identifying the preparatory phase of large earthquakes and mitigate seismic risk.

## Introduction

On 6 February 2023, at 01:17:34, a strong earthquake of Mw 7.8 struck the region between Türkiye and Syria with the epicenter near the city of Kahramanmaraş, Türkiye^1^ (hereinafter, Mw 7.8 EQ). Just 9 h after, another strong earthquake (Mw 7.5) struck the same region on the Sürgü-Misis-Fault with the epicenter near the city of Ekinözü, Türkiye^2^. These two earthquakes, and the consequent aftershock sequence caused near 60,000 fatalities and more than 100,000 injured. The Anatolian region is characterized by a counterclockwise movement and exhibit a rigid block behavior with the main deformations that appear on the North Anatolian Fault (NAF) and on the East Anatolian Fault (EAF)^3,4^, which is mainly characterized by a sinistral strike-slip behavior. The 2023 sequence took place in the EAF, a fault system approximately 580 km long and which ranges from the triple junction of Karlıova (Eastern Türkiye) going in the Southwest direction to Antakya near the Mediterranean Sea boarding Türkiye-Syria border^4–6^. Geological and geomorphological studies show the slip-rate varying significantly along the EAF system, with a maximum of 10 mm·year^−1^ near Karlıova in the east to 2.5 mm·year^−1^ in the west^7,8^. Here, we consider, from east to west, the five EAF fault segments of Palu, Pütürge, Erkenek, Pazarcık and Amanos^8^. The historical and instrumental seismicity of the area is well documented^9–11^ and the Mw 7.8, 6 February 2023 Kahramanmaraş earthquake is the strongest event ever happened on the EAF in historical record; eventually comparable with the 995 Palu earthquake, which magnitude, however, remains uncertain^9,11^. It is worth to note that the 2023 earthquakes filled a well-known gap in the seismicity^8–10,12^, but the high magnitude of the event was unexpected considering the historical seismicity of the area. The Mw 7.8 Kahramanmaraş earthquake initiated on a small fault extending southwestward from the main branch of the EAF^13^, and then developing into a bilateral rupture that involved at least the Amanos, Pazarcık and Erkenek segments^1,14^.

For the Amanos and Pazarcık segments, Güvercin et al.^8^ estimated a maximum magnitude M_max_ equal to 7.4 every 772–915 years. On the other hand, looking at historical stress accumulation, Nalbant et al.^12^ stated that the Pazarcık segment was “the most likely location of the next damaging earthquake on the EAF … likely to produce a very large (Mw ≥ 7.3) event”.

**Figure 1:**
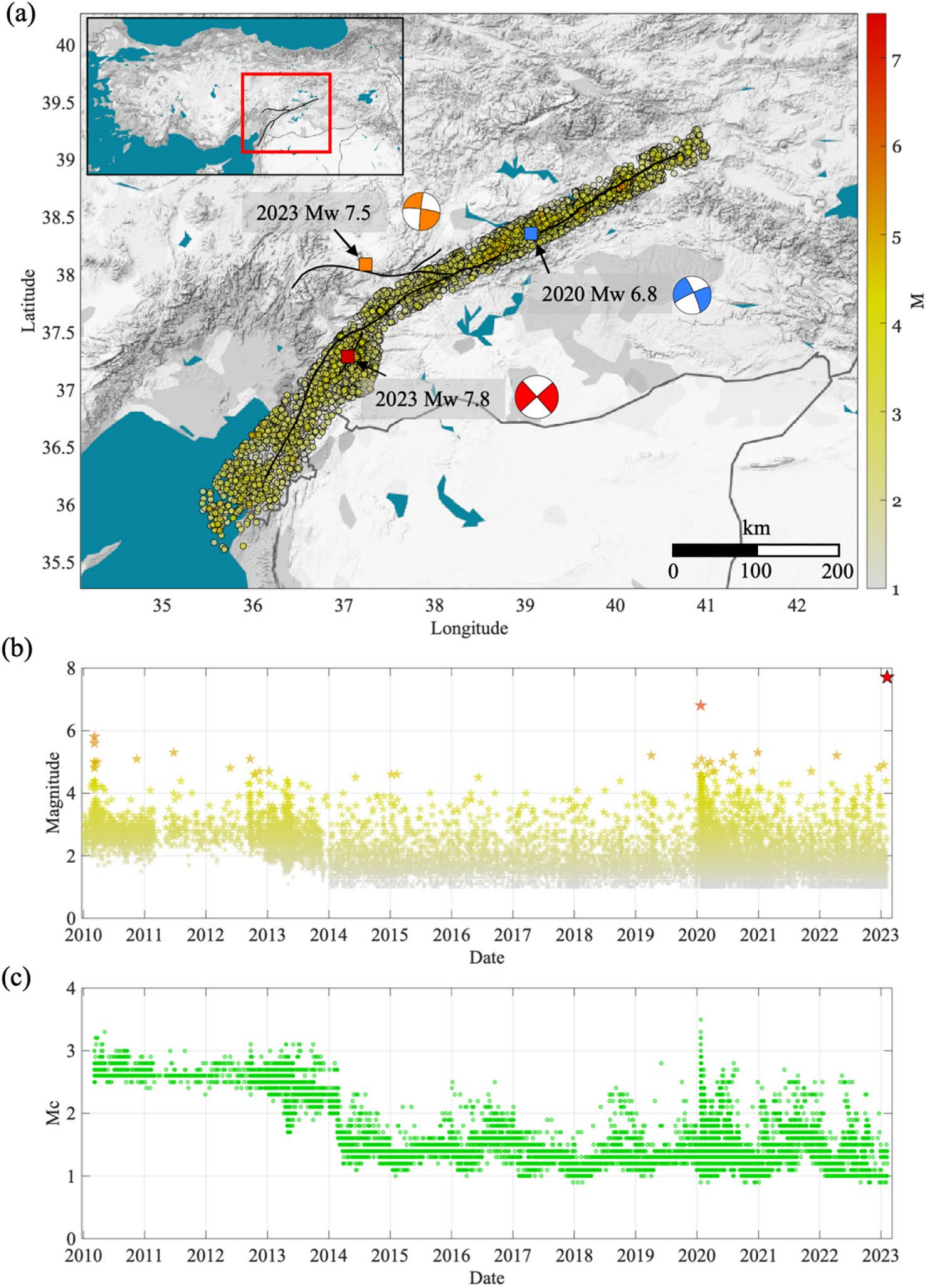
Location and magnitude distribution of the seismicity. (**a**) Location of the earthquakes (circles colored per magnitude), the epicenter of the 2023 Mw 7.8 Kahramanmaraş earthquake (red square), the one for the 2023 Mw 7.4 Ekinözül earthquake (orange square), and the one for the 2020 Mw 6.8 Elazığ earthquake (blue square). The focal mechanisms for the three main earthquakes are shown with the same color as the epicenters. The outline of surface rupture is shown as black lines^14^. (**b**) Distribution of magnitude in time. The events are colored per magnitude as in (**a**), while the 2023 Mw 7.8 earthquake is highlighted in red. (**c**) Temporal evolution of the completeness magnitude Mc. The map was done using Matlab software (R2019b, https://it.mathworks.com/, last accessed September 2023).

According to the USGS bulletin, the Mw 7.8 EQ presents an almost vertical pure strike-slip focal mechanism (i.e., strike 318°, dip 89°, and rake − 179°)^1^. In a very short time after the Mw 7.8 earthquake, many studies have been published regarding the source characteristics^14–19^. Results from different techniques, such as back-projection and remote sensing^14^, consistently point out that the Mw 7.8 mainshock originated from an unmapped structure connected to the Pazarcık segment (Fig. 1). Then, the rupture propagated to the Pazarcık segment and proceeded first northwards on the Pazarcık and Erkenek segments and then southwards on the Amanos segment (Supplementary Fig. S1)^14,16,18^.

Looking at the recent past seismic activity in the region, the most significant previous earthquake along the EAF was the January 2020 Mw 6.8 Elazığ earthquake^8,20–23^, which took place on the Pazarcık segment. The latter earthquake was mainly a left-lateral strike-slip, with a small portion of source slip presenting a down-dip behavior^22^. It is worth noting that Xu et al.^22^ recognized in this event a new activation of the EAF and they warned the seismic hazard community to give particular attention to the seismic gaps previously indicated by Nalbant et al.^12^. Of outmost importance, Güvercin et al.^8^ showed that the 2020 Mw 6.8 Elazığ earthquake “was preceded by an accelerated seismic activity” started one month before with a Mw 5.2 event on the same fault. Moreover, Konca et al.^23^ showed that the aftershocks remained on the area of the mainshock for three months after they started to migrate southwards.

The existence of a one-month preparatory process for the 2020 Mw 6.8 Elazığ earthquake stimulates us to investigate the existence of generation processes for the 2023 Mw 7.8 Kahramanmaraş earthquake. Studies aiming at identifying a preparatory phase for the latter earthquake has been recently carried out focusing on the evolution and properties of seismic clusters within few tens of kilometers from the Kahramanmaraş earthquake epicentre^24^ and on the spatio-temporal variation of the Gutenberg-Richter *b*-values at regional scale^25^.

Retrospective studies of past large earthquakes^26–30^, especially megathrusts, are showing us that main ruptures are often anticipated by preparatory processes, though their identification is difficult because the precursory patterns vary depending on the tectonic environment^30^ (e.g., quiescence, foreshocks, accelerating seismic release, doughnut, and event migration inward and outward the nucleation area, slow slip events and creep phenomena identifiable by geodetic measurements).

Differences in the preparatory phase of large earthquakes highlight that crustal processes leading to them are influenced by unforeseeable combination of heterogeneous fault properties, stress interactions and crustal fluids. Such complexity lets the background physical processes generating large earthquakes not fully understood yet. Despite, how, when, and where large earthquakes are generated remain fundamental unsolved scientific questions, studying the preparatory phase represents a challenge that the seismological community is called urgently to address to mitigate seismic risk.

Promising observations come from patterns in small magnitude seismicity and crustal deformation preceding larger earthquakes^28,32–35^. Kato and Ben-Zion^31^ made a review of decades of observational, laboratory and theoretical studies, which leads them to suggested large earthquakes being generated by a progressive localization of shear deformation around a rupture zone that progressively evolves into a final rapid loading of a crustal volume localized nearby the hypocenter of the major dynamic rupture. During the latter process, small magnitude earthquakes, commonly called foreshocks, are generated, and their pattern and peculiar dynamic characteristics^36,37^ might be the key for identifying the preparatory phase of large earthquakes. Similar patterns in damage evolution have been observed by studying acoustic emissions during triaxial tests on rock samples^38^, suggesting that the process generating earthquakes may be universal.

As it is currently impossible to identify whether an earthquake is a foreshock or not, and such label is assigned to events retrospectively only, we focus our analyses on the spatio-temporal evolution of earthquakes with magnitude larger than M_L_ 1.5 that occurred along the main segments of the East Anatolian Fault (EAF) since 2014. We analyze the spatiotemporal clustering and physical based features describing different aspects of the seismicity evolution seeking for clues of stress accumulation^32^ and/or transfer among EAF segments, and changes in coupling conditions^39^. Observing the evolution in the proportion of nonclustered seismicity, which is interpreted as a proxy for coupling^39,40^, we find different temporal patterns and insights of a progressive decoupling process interesting both the Pütürge segment before the 2020 Mw 6.8 Elazığ earthquake and the Pazarcık one before the Mw 7.8 EQ. The preparatory phase of the Mw 7.8 EQ is also identified by the temporal evolution of b-value, fractal dimension and energy rate. Observing these seismic features, which can be considered as proxy for the crustal stress^32^, we can identify a first long-term trend in the earthquake characteristics for the Amanos and Pazarcık fault segments following the 2020 Mw 6.8 Elazığ earthquake on the Pütürge segment that can be interpreted as quiescence. The latter was followed by a final stage in the preparatory process lasting ~ 8 months before the Mw 7.8 EQ.

## Results

### Evolution of clustered and non-clustered seismicity before the 2023 Mw 7.8 earthquake

We selected the seismicity that occurred along the EAF region since 2010 and until the Mw 7.8, 6 February 2023 earthquake. The available catalog includes ~ 17,000 earthquakes with magnitude larger than M_L_ ≥ 1 within a buffer of variable width between 30 and 50 km from east to west of the EAF, respectively (Fig. 1a). Earthquakes’ catalog information is retrieved by the “Disaster and Emergency Management Authority of the Republic of Türkiye”^41,42^ (i.e., Event ID, origin date and time, Longitude, Latitude, Depth and Magnitude). The latter considers the local magnitude (M_L_) for earthquake smaller than about M_L_ 4 and the moment magnitude (M_w_) for the larger ones. The temporal evolution of magnitude suggests inhomogeneous completeness for the years before 2013 (Fig. 1b). We verify the time dependency of the magnitude of completeness (Mc, see “Methods”) and we observe an abrupt change in 2014 (Fig. 1c). For the period 2014–2023, Mc varies mainly between M_L_ 1 and 1.5 (mode M_L_ 1.3). We thus select for the following analyses the earthquakes occurred in the period 2014–2023 and magnitude M_L_ ≥ 1.5, for a total of 7502 events (Supplementary material S1).

**Figure 2:**
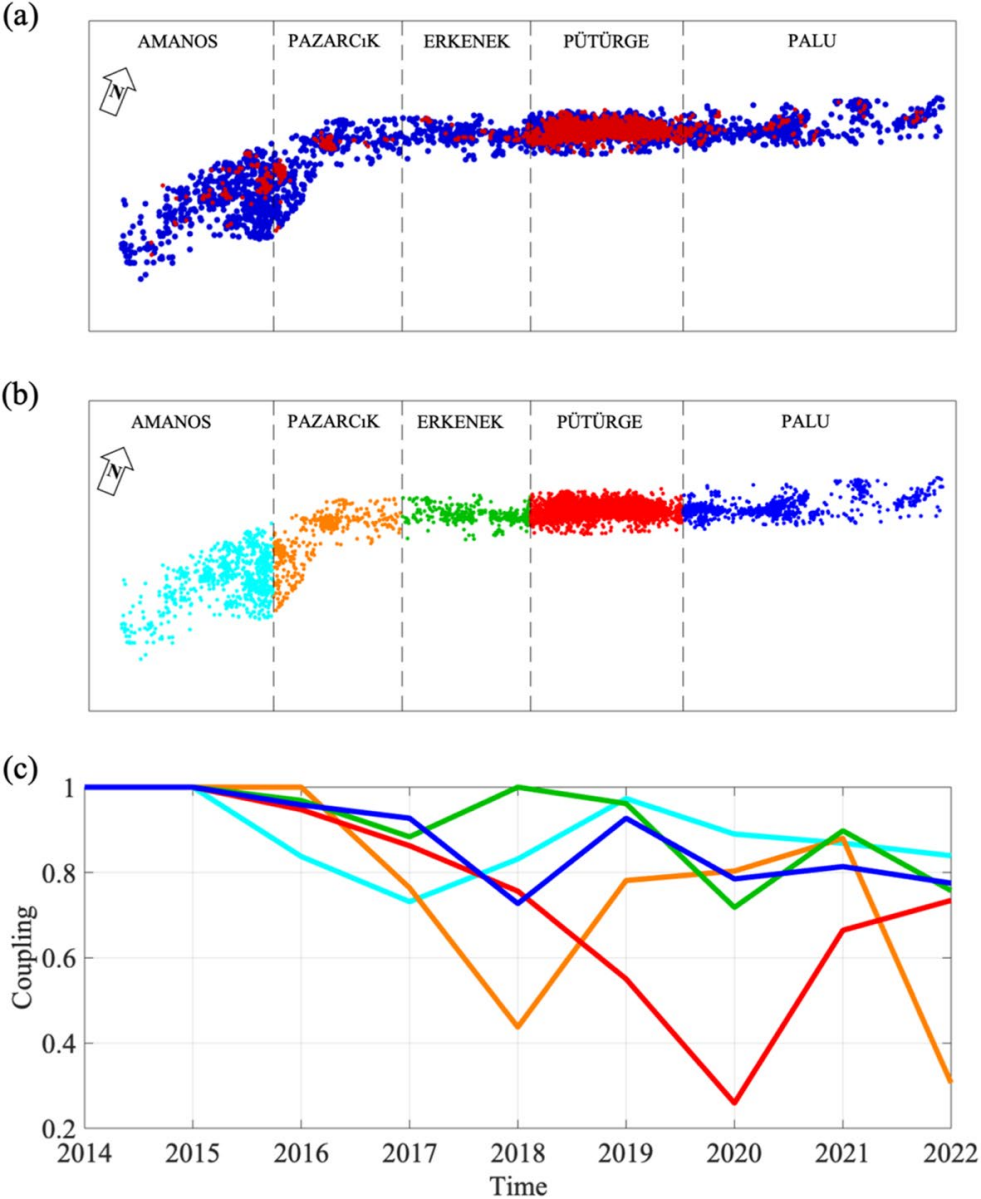
Nearest-neighbor distance approach applied to the EAF seismicity. (**a**) Distribution of earthquake epicenters (background events in blue and clustered events in red). Boundary of the EAF's fualt segments^8^ (black dashed lines), North direction is indicated on the top left. (**b**) Distribution of earthquakes for the EAF’s fault segments. (**c**) Temporal evolution of the fraction of non-clustered seismicity, which is assumed being a proxy of coupling, for each EAF’s fault segment (Lines are colored following subplot b).

We apply the nearest-neighbor approach^43^ to isolate clusters from background seismicity (Fig. S1; see “Methods”). To this aim, we rely on a Gaussian mixture distribution model to split the generalized distance, η, distribution in clustered and background seismicity. As discussed by Aden-Antóniow et al.^44^, by doing this we accept the risk that the two populations slightly contaminate each other. In the period 2014–2023, the 2020 Mw 6.8 Elazığ earthquake represents the only event of large magnitude before the Mw 7.8 EQ. Hence, we perform the nearest-neighbor analysis considering three time periods: (i) from 2014 to 2019, (ii) 2020, and (iii) from 2021 to 2023. Periods (i) and (iii) presents very similar η distribution, with a clear distinction between clustered and background seismicity (Fig. S2), while period (ii) appears strongly dominated by clustered seismicity. The spatial distribution of clustered and background seismicity depicts a clear inhomogeneous pattern (Fig. 2a), where the Pütürge segments presents the highest clustered events following the 2020 Mw 6.8 Elazığ earthquake with apparently very limited effects on the near Palu and Erkenek segments. Previous results concerning the spatio-temporal evolution of the seismicity have already highlighted that the EAF is highly heterogeneous and segmented, where fault segments poorly interact each others^8^. Therefore, we develop our following analyses considering the five main segments of the EAF separately (i.e., from east to west, Palu, Pütürge, Erkenek, Pazarcık and Amanos^8^, Fig. 2b).

We compute for each segment and each year between 2014 and 2023 the fraction of non-clustered seismicity (see “Methods”), which we interpret as a proxy for heterogeneity and fault coupling^39,40^. It is worth mentioning the significant differences in the number of earthquakes associated with each segment: 796 events for Amanos, 494 for Pazarcık, 234 for Erkenek, 5256 for Pütürge, and finally 722 for Palu. The Amanos, Erkenek, and Palu segments show temporal variation of coupling but of rather limited amplitude (Fig. 2c). We find interesting the progressive, long-lasting decrease in coupling for the Pütürge segments, which culminated with the Mw 6.8 Elazığ earthquake in January 2020. Then, the coupling of the latter segment seems to recover in the following years. It is worth noting that studies carried out after the 2020 Elazığ earthquake considered the Pütürge segment as heterogeneous and partially coupled^45^. Even the Pazarcık segment shows temporal variations. The low coupling level achieved in 2018 does not correspond to large earthquake magnitudes in any segments (only a couple of events with magnitude around Mw 4.5 are identifiable for the Pazarcık segment, Fig. S3). Instead, we find interesting the decrease in coupling shown by Pazarcık segment in the 2022, which we know was followed by the Mw 7.8 EQ. Below, we test whether the observed temporal changes in the non-clustered seismicity fraction have a counterpart in terms of stress-related seismic features.

### Temporal evolution of seismic features

**Figure 3:**
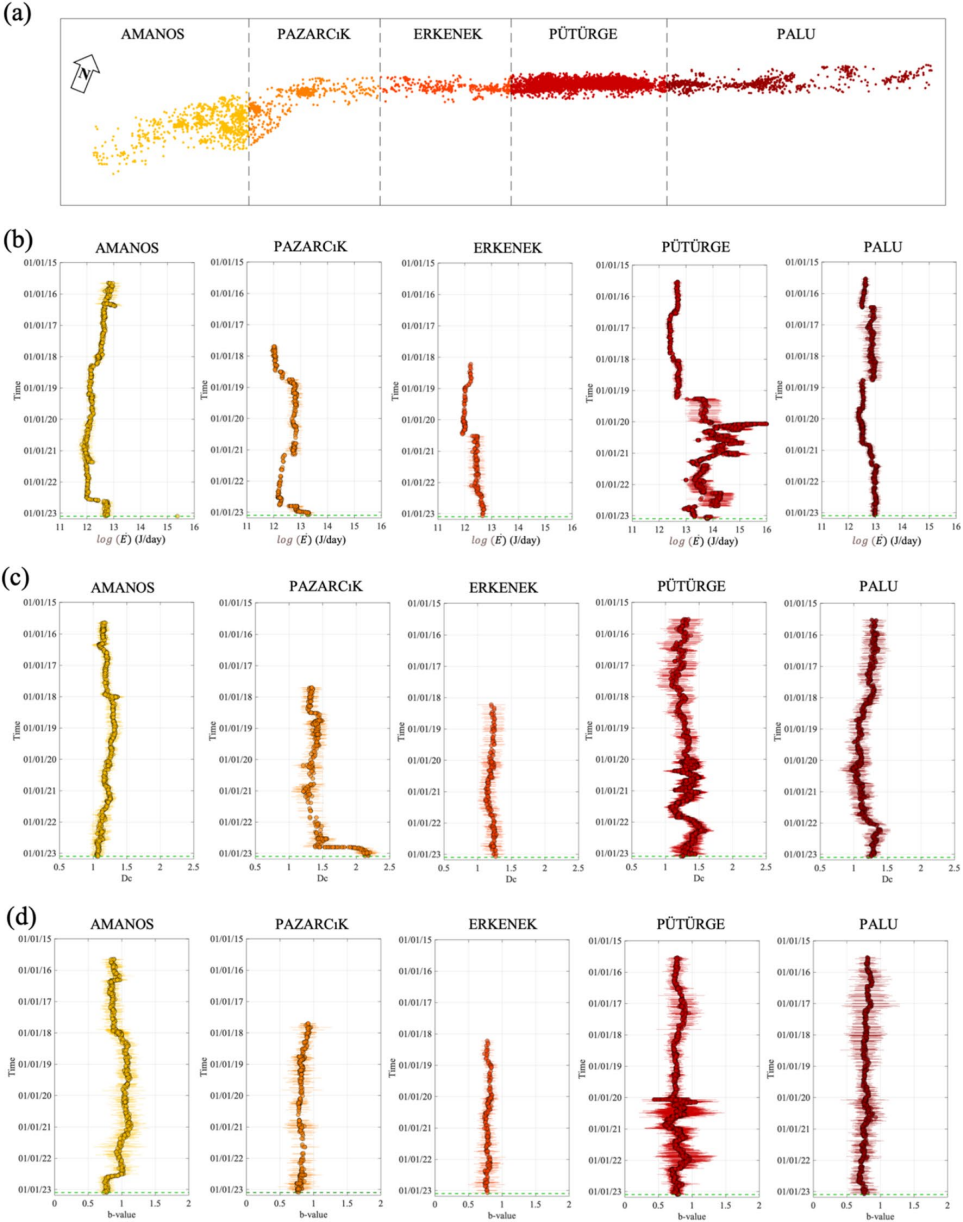
Seismic features for the EAF’s segments. (**a**) Distribution of earthquakes colored for the EAF’s fault segments. (**b**) Temporal evolution for the logarithm of the energy rate, $\dot{{\varvec{E}}}$. We show the mean value ± the standard error (horizontal bar). (**c**) The same as (**b**), but for the fractal dimension, Dc. (**d**) The same as (**b**), but for the b-value.

We characterize the seismicity in terms of features describing different aspects of the temporal evolution of seismicity: the energy rate^46^($\dot{E}$), the Gutenberg-Richter *b*-value^47^ and the fractal dimension^48^ (Dc). The analysis is performed by considering moving time-windows of 100 events, and we move them of one event at a time (see “Methods”)^32,49^. The uncertainty associated to the features is estimated by applying a bootstrap approach^50^, that is by repeating at each time instant the features computation with 200 random sampling realizations of the original dataset with replacement. The temporal evolution of $\dot{E}$, Dc and *b*-value for each segment is shown in Fig. 3. The first striking evidence in our results is the abrupt change in the $\dot{E}$ trend for the Pütürge fault in 2019, to which follows the further energy rate increase in occasion of the Mw 6.8 Elazığ earthquake in January 2020 (Fig. 3b central panel). However, it is worth noting that any clear change is visible during the 2019 for *b*-value and Dc. The Erkenek and Palu segments next to Pütürge show no trend in any features, which agrees with our results concerning the coupling evolution and previous studies^8^. The second clear evidence is the characteristic change in trend for the analyzed features for the Amanos and Pazarcık segments. In Amanos, we see a clear decrease in *b*-value and the increase of $\dot{E}$ e few months before the Mw 7.8 EQ. In Pazarcık, we see $\dot{E}$ decreasing since 2021 and then increasing as in Amanos. Furthermore, we observe even the increase in Dc.

**Figure 4:**
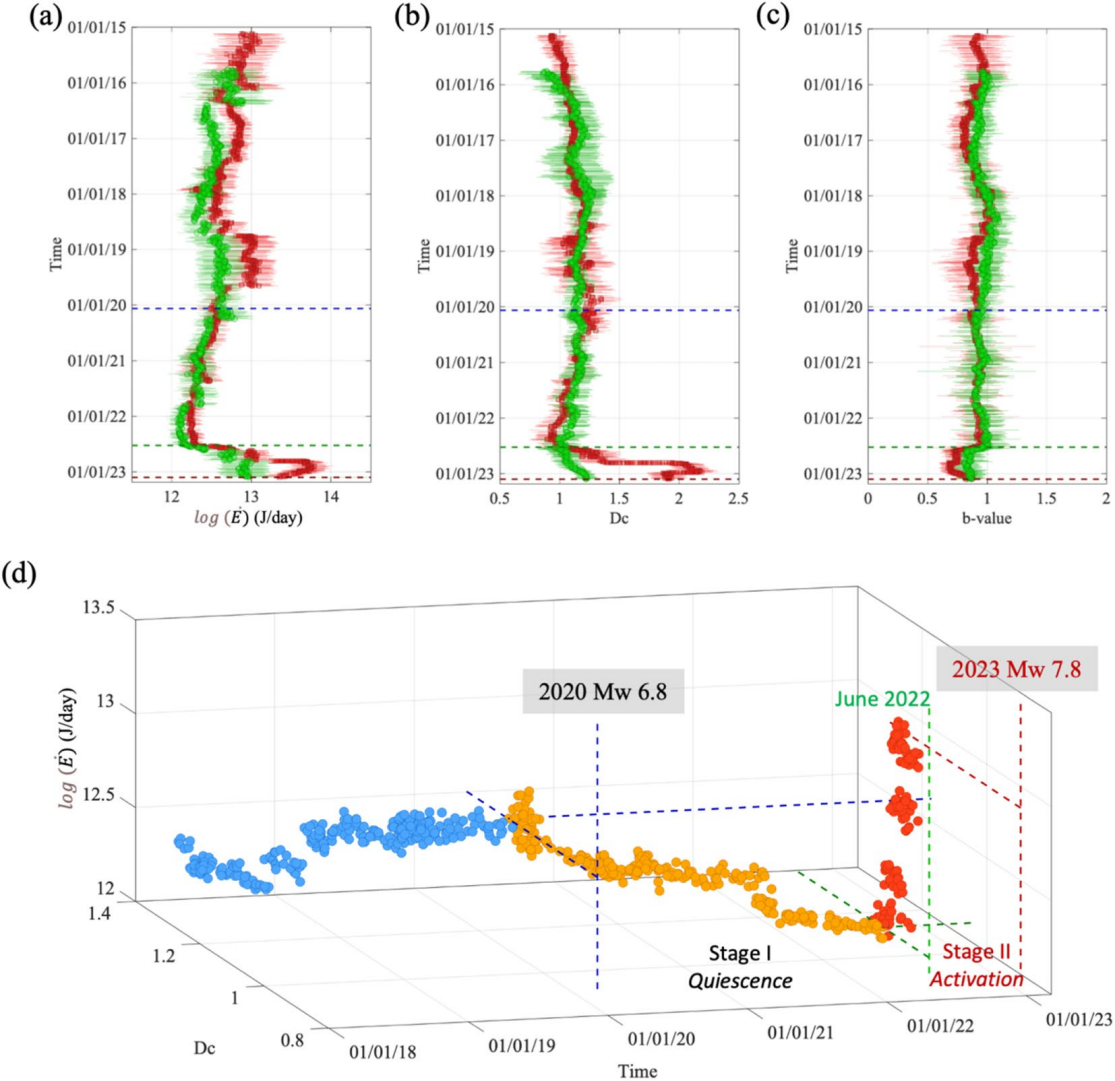
Temporal evolution of seismic features for the Amanos and Pazarcık segments combined. (**a**) Temporal evolution for the logarithm of the energy rate, $\dot{{\varvec{E}}}$ for clustered (red dots) and background (green dots) seismicity. We show the mean value ± the standard error (horizontal bar). (**b**) The same as a, but for the fractal dimension, Dc. (**c**) The same as a, but for the b-value. (**d**) Temporal evolution of $\dot{{\varvec{E}}}$ and Dc colored for different time periods: before the 2020 Mw 6.8 Elazığ earthquake (blue), between the latter and ~ 8 months before the mainshock (yellow), and for the ~ 8 months (red). The boundaries of the time periods and the average level of features at these time intervals are shown as colored dashed lines.

Since the Mw 7.8 EQ nucleated from an unmapped structure connected to the Pazarcık segment at the boundary with the Amanos segment (the area corresponds to the Türkoglu releasing step-over^6^), we combine the datasets for the two segments and calculate the seismic features considering: (i) all the events, (ii) only the background seismicity. Although there are some differences between the two datasets (Fig. 4, with seismic features for background only and all the events are shown in green and red, respectively), the main changes in the temporal evolution of $\dot{E}$, Dc and *b*-value persist. We mark in Fig. 4 the origin time of the Mw 6.8 Elazığ earthquake in January 2020 (blue dashed line) and the moment when we see a significant change in the trend of the features (i.e., ~ 8 months before the Mw 7.8 EQ, green dashed line). In the interval between these two instants, a progressive decrease in both $\dot{E}$ and Dc is observed (Fig. 4a,b), while no change is observed for the *b*-value (Fig. 4c). During the ~ 8 months preceding the main event, we observe the increase of both $\dot{E}$ and Dc, and a decrease in *b*-value, no matter what kind of seismicity we consider.

## Discussion

The evolution of seismicity along the EAF in the years preceding the Mw 7.8 Türkiye EQ shows peculiar spatio-temporal patterns. Following Güvercin et al.^8^, we performed our analyses considering the main fault segments involved during the Mw 7.8 EQ. The fraction of non-clustered events indicates heterogeneous fault segments characterized by different level of coupling and response to stress perturbations. Differences in coupling between fault segments, and even within single one, where previously reported by both seismological and geodetic information^8,45^. According to previous studies^51^, the intermediate seismic coupling of the EAF and the temporal distribution of the past large earthquakes have led to the possibility of large earthquakes being considered probable. Our results show temporal changes in fault properties for the Pütürge and Pazarcık segments before the Mw 6.8 Elazığ earthquake in 2020 and in the year before the Mw 7.8 EQ in 2023, respectively. Time dependencies in segment coupling suggest that both these segments were getting closer to instability driven by a long-lasting process.

Our analysis was then directed to the study of the features that describe different aspects of the temporal evolution of seismicity. For the Pazarcık and Amanos segments, the considered features present a clear change in trend starting ~ 8 months before the Mw 7.8 EQ in 2023, in agreement with recent studies that analyzed the clustering of seismicity around the mainshock epicentral area^24^ and the spatio-temporal variation of the Gutenberg-Richter *b*-values at regional scale^25^. The decrease in *b*-values is coherent with the rising of $\dot{E}$, being both these features related to crustal stress. The fractal dimension Dc has been reported to vary before large earthquakes in different tectonic context^52,53^. We therefore look at the temporal evolution of $\dot{E}$ and Dc together for the Pazarcık and Amanos segments aiming at qualitatively capturing the main patterns in the preparatory phase of the Kahramanmaraş earthquake (Fig. 4d). The effects of the Elazığ earthquake in 2020 on the Pazarcık and Amanos were not clearly detected in previous studies^8,54^. The Coulomb stress failure associated to the Elazığ earthquake was estimated to be negligible and not able to advance ruptures on the Pazarcık segment^54^. Nevertheless, the long-lasting phase characterized by a decrease in both $\dot{E}$ and Dc that started on the Pazarcık segment soon after the Elazığ earthquake (Fig. 4d), let us to hypothesize that even the small change stress change generated by the 2020 earthquake promoted the beginning of the long preparation phase for the Kahramanmaraş earthquake. The period 2020–2022 might therefore correspond to a quiescence phase for the Kahramanmaraş earthquake (stage I, Fig. 4d). Further studies about the role of historical earthquakes in altering the stress field of the EAF segments, as for instance the one of Chen et al.^55^, are necessary to confirm our hypothesis. The activation phase (stage II, Fig. 4d) started with the background seismic activity changing its topological dimension (i.e., change in Dc) and accelerating capacity to release the accumulated strain energy (i.e., increase in $\dot{E}$), causing a gradual unpinning of the fault where the Kahramanmaraş earthquake nucleated. It is also worth noting that the latter activation phase did not start after a moderate-large earthquake (Fig. S3), in contrast with those of other recent large earthquakes such as, the 2009 L’Aquila earthquake^36^, the 2011 Tohoku-oki megathrust^31^ or the 2016 Kumamoto earthquake^31^.

Our results highlight that the spatio-temporal evolution and dynamic characteristics of small magnitude earthquakes can be important information for updating the hazard in areas with high seismic potential, as also shown for moderate magnitude induced earthquakes^49^. Systematic studies on the properties of the earthquakes’ source and their temporal evolution could also be useful to shed light on the dynamic characteristics and healing of microcracks^56,57^ during the preparation phase. The latter could in fact help to grasp clues that a major rupture is near. Further complementary information about the nature of the preparatory process can be derived by geodetic data. It would be relevant, in fact, to investigate if the Kahramanmaraş earthquake was promoted also by an aseismic process.

## Methods

### Seismic data and features

We rely on seismic data from the “Disaster and Emergency Management Authority of the Republic of Türkiye” catalog^41^. The seismic catalog includes the information: event ID, origin date and time, Longitude, Latitude, Depth and Magnitude. We include the used catalog as ‘Supplementary_S1_catalog_R1.csv S1’. The magnitude of the earthquakes range between the local magnitude M_L_ 1.5 and the moment magnitude M_w_ 7.8.

An in deep analysis of the AFAD catalog characteristics is provided by Çıvgın and Scordilis^42^, to which we refer for a comparison with those provided by KOERI and ISC.

We extract seismic features characterizing the evolution of seismicity by considering windows of events with fixed length (100 earthquakes) and we move them of one event at time. The value of each feature is assigned to the last event in a window. The seismic features (Date and time of the last event in a time window, Longitude and Latitude for the center of mass, rate, energy rate and energy per event) are computed for each EAF’s segment and included in the supplemental material (Supplementary_S2_Amanos_R1.csv S2, Supplementary_S3_ Pazarcık_R1.csv S3, Supplementary_S4_Erkenek_R1.csv S4, Supplementary_S5_Pütürge_R1.csv S5, Supplementary_S6_Palu_R1.csv S6).

### Seismic features

(i) The b-value is estimated by analyzing the frequency-magnitude distribution by the Gutenberg–Richter law^47^

1 $$ {\text{logN }} = {\text{ a }}{-}{\text{ b}} \cdot {\text{Mw}}, $$

where N is the cumulative number of earthquakes, a and b values are parameters describing the productivity and relative event size distribution). The b value and the magnitude of completeness Mc are estimated by the software package ZMAP^58^ applying the maximum likelihood approach^59^ and the maximum-curvature method^60^.

(ii) The energy rate $\dot{{\varvec{E}}}$ is computed similarly to what is done for the moment rate^46^. Estimates of the radiated energy of earthquakes E is derived from the local magnitude M_L_ of the earthquake^61^ (i.e., E = 1.96 M_L_ + 2.05; where E is in J).

Therefore, $\dot{{\varvec{E}}}$ is computed as follows:

2 $$\dot{{\varvec{E}}}=\lambda EA\frac{b}{1.5-b}\left[{10}^{(1.5-b)({m}_{max}-{m}_{0})}-1\right],$$

where λ is the seismic rate of events larger than Mc, b is the b-value, m_*max*_ and m_0_ correspond to the largest magnitude and to the Mc in the considered time window, A is the area of finite extension including the events (in km^2^). The seismic rate λ is obtained considering the number of events ΔN with magnitude larger than the completeness magnitude, Mc, that occurred in a time window ΔT in areas of finite extension A. The latter is computed as the convex Hull of the epicenters in each time window. The seismic rate is thus computed as follows:

3 $$\gamma =\frac{\Delta N}{(\Delta T\cdot A)},$$

where λ represents the events per day per square kilometers [eqks./(day·km^2^)].

### Nearest-neighbor distance, η, and fraction of nonclustered seismicity

The nearest-neighbor approach^43^ computes the generalized distance between pairs of earthquakes, η, by an analysis of the time–space distances between pairs of earthquakes. η is obtained by estimating the distances in time (i.e., rescaled time, T_η_) and space (i.e., rescaled distance, R_η_) between an event *i* and its parent *j*, where both distances are normalized by the magnitude of the parent event. The rescaled time and distance are computed as follows:

4 $${T}_{ij}={t}_{ij}{10}^{-b{m}_{i}/2},$$

5 $${R}_{ij}={\left({r}_{ij}\right)}^{{D}_{c}}{10}^{-b{m}_{i}/2},$$

where, *m* is the magnitude, b is the parameter of the Gutenberg–Richter law, which plays the role of exponential weight of the earlier event *i* by its magnitude, and D_c_ is the fractal dimension. Finally, η is defined as:

6 $$log{\eta }_{ij}= log{R}_{ij}+ log{T}_{ij},$$

We compute η considering the epicentral location of the earthquakes. According to Zaliapin and Ben-Zion^62^, we set the b equal to 0 to mitigate the presence of artifacts due to the overlap of earthquakes’ domain of attraction with background seismicity, and we use D_c_ equal to 1.5.

We model the η distribution with a sum of a log-Gaussian function^44^ and we split the earthquakes population in clustered (C) and background (B) seismicity. Then, the proportion of nonclustered seismicity (P_NS_) is estimated for each fault segment and year as the ratio between the number of events (n) belonging to B and those of both C and B: P_NS_ = nB/(nB + nC).

### Supplementary Information

Supplementary Figures.

Supplementary Information 2.

Supplementary Information 3.

Supplementary Information 4.

Supplementary Information 5.

Supplementary Information 6.

Supplementary Information 7.

## Data Availability

We used data and information retrieved from the Republic of Turkey—Ministry of Interior Disaster and Emergency Management Authority (AFAD) from https://deprem.afad.gov.tr/event-catalog. Supplemental material includes three figures and six files, which are named: ‘Supplementary_S1.csv’; the seismic features for the EAF’s segments, are included in the files: ‘Supplementary_S2.csv’ for Amanos, ‘Supplementary_S3.csv’ for Pazarcık, ‘Supplementary_S4.csv’ for Erkenek, ‘Supplementary_S5.csv’ for Pütürge, ‘Supplementary_S6.csv’ for Palu.
