# Supplementary material for: The preparatory process of the 2023 Mw 7.8 Türkiye earthquake
Source: Sci Rep. 2023 Oct 19;13:17853. doi: 10.1038/s41598-023-45073-8 (PMC10587168; doi:10.1038/s41598-023-45073-8)
Supplement: Supplementary file 1 — Supplementary Figures. [file 41598_2023_45073_MOESM1_ESM.docx]

Supplementary Materials for

The Preparatory Process of the 2023 Mw 7.8 Turkey Earthquake

Matteo Picozzi*, Antonio G. Iaccarino and Daniele Spallarossa

*Corresponding author. Email: matteo.picozzi@unina.it

**This file includes:**

This file includes 3 figures, from Fig. S1 to Fig. S3.


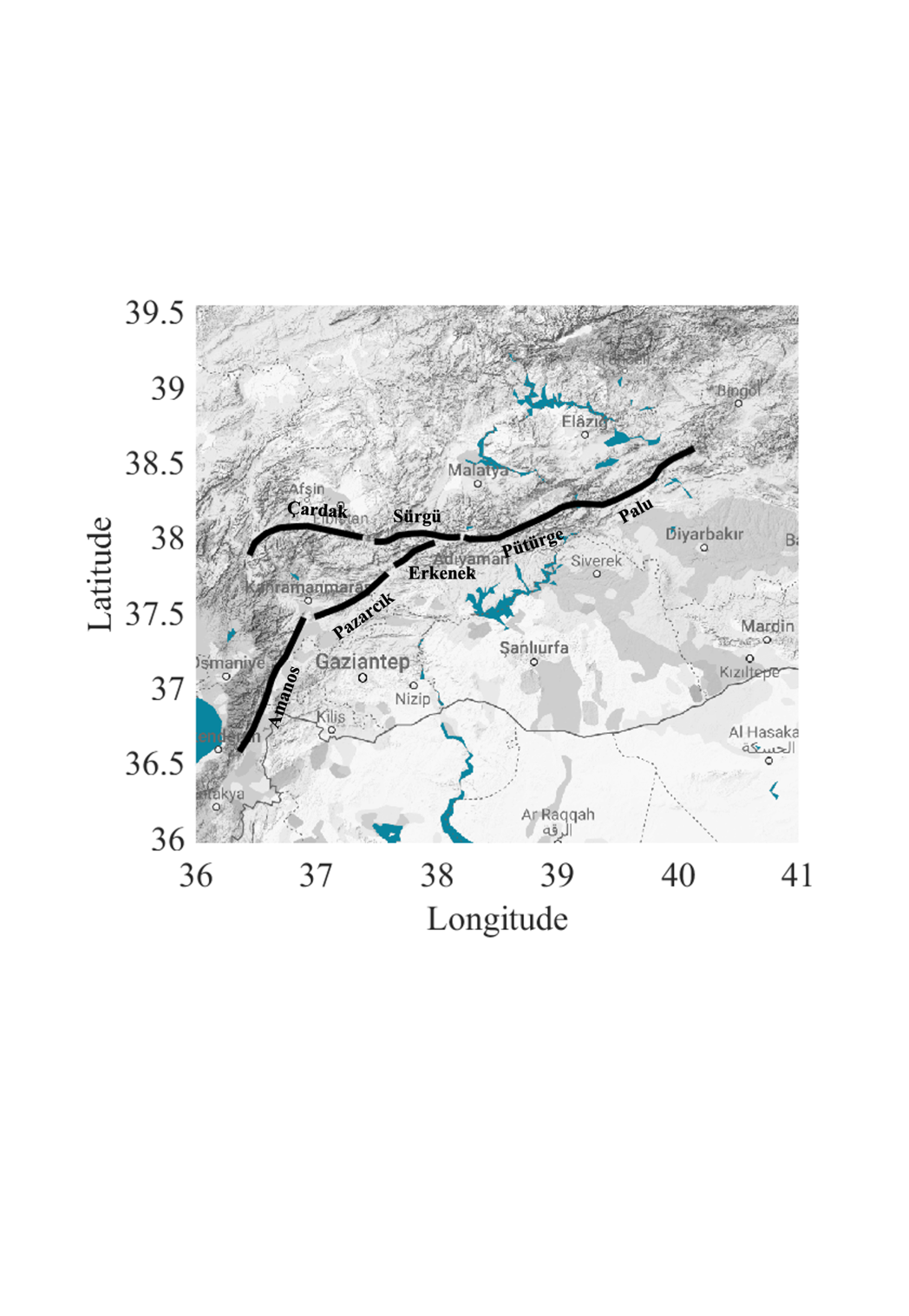


**Figure S1.** Distribution of EAF’segmennts involved in the 2023 Mw 7.8 Kahramanmaraş earthquake, the 2023 Mw 7.4 Ekinözül and the 2020 Mw 6.8 Elazığ earthquakes.

**
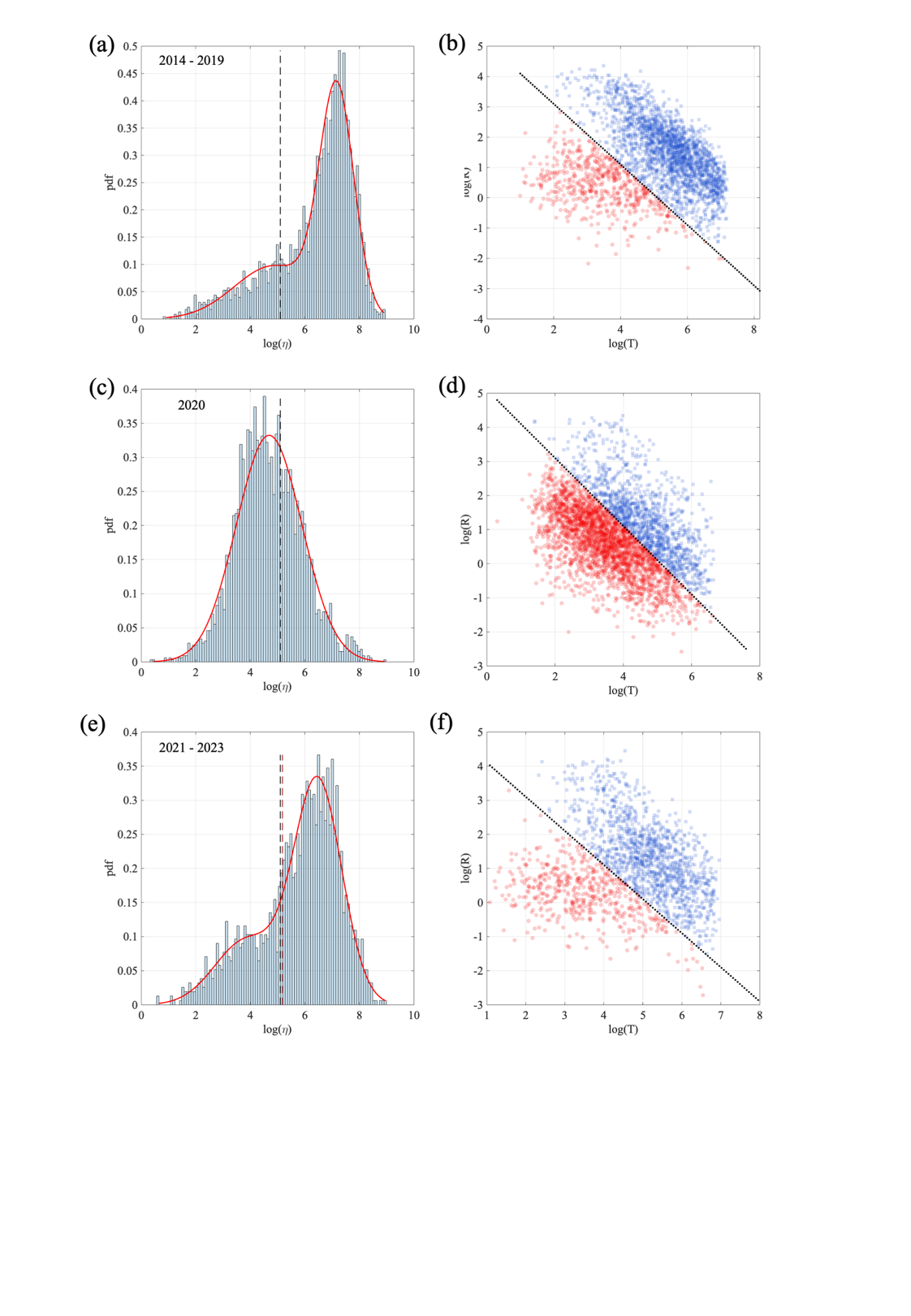
**

**Figure S2.** Results of the nearest-neighbor approach^43^. **a.** Generalized distance distribution for earthquakes occurred between 2014 and 2019, Gaussian mixture distribution model (red line), generalized distance threshold, η, used to split the distribution in clustered and background seismicity (dashed black line). **b.** Rescaled time T and distance R of the nearest-neighbor distance 𝜂 for clustered (red) and background (blue) seismicity. η threshold splitting the distribution in clustered and background seismicity (dotted black line). **c.** and **d.** The same as a and b, respectively, but for the seismicity occurred during the 2020. **e.** and **f.** The same as a and b, respectively, but for the seismicity occurred between the 2021 and 2023. [^43.^ Zaliapin, I., Gabrielov, A., Keilis-Borok, V. & Wong, H. Clustering analysis of seismicity and aftershock identification. Phys Rev Lett 101, (2008)].


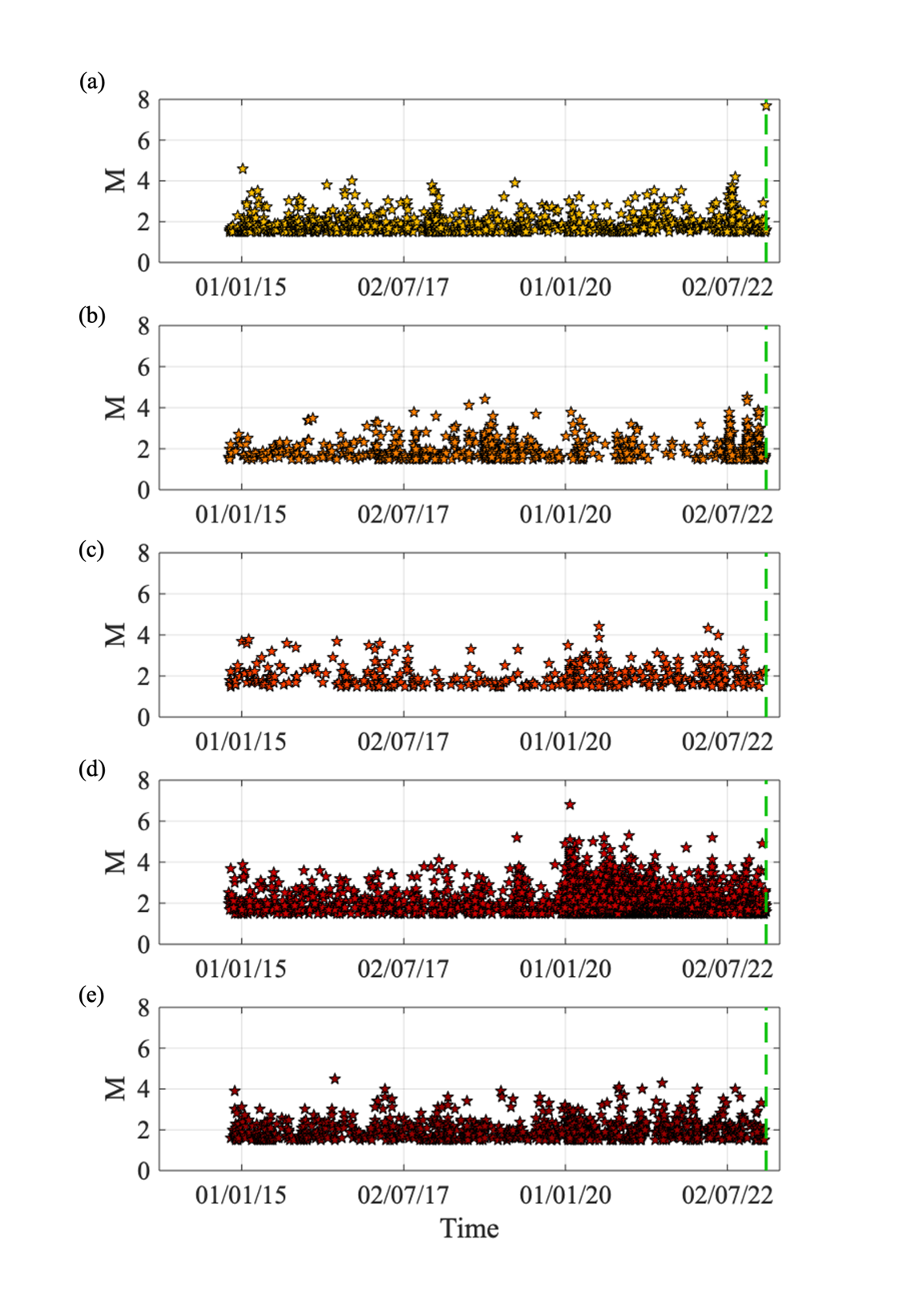


**Figure S3.** Temporal evolution of magnitude for the EAF’s fault segments. a. Seismicity for the Amanos segment. b. as for a., for Pazarcık. c. as for a., for Erkenek. d. as for a., for Pütürge. e. as for a., for Palu. The occurrence of the 2023 Mw 7.8 Kahramanmaraş earthquake is indicated by a green dashed line.
